# *Streptococcus pyogenes*-purpura fulminans as an invasive form of group A streptococcal infection

**DOI:** 10.1186/s12941-018-0282-9

**Published:** 2018-07-09

**Authors:** Sayaka Okuzono, Masataka Ishimura, Shunsuke Kanno, Motoshi Sonoda, Noriyuki Kaku, Yoshitomo Motomura, Hisanori Nishio, Utako Oba, Masuo Hanada, Jun-ichi Fukushi, Michiyo Urata, Dongchon Kang, Hidetoshi Takada, Shouichi Ohga

**Affiliations:** 1 Department of Pediatrics, Graduate School of Medical Sciences, Kyushu University, 3-1-1 Maidashi, Higashi-ku, Fukuoka, 812-8582 Japan; 2 Division of Pediatrics, Oita Prefectural Hospital, Oita, Japan; 3 Department of Plastic Surgery, Kyushu University Hospital, Fukuoka, Japan; 4 Department of Orthopaedic Surgery, Graduate School of Medical Sciences, Kyushu University, Fukuoka, Japan; 5 Department of Clinical Chemistry and Laboratory Medicine, Kyushu University Hospital, Fukuoka, Japan

**Keywords:** Acute infectious purpura fulminans, Invasive group A β-Streptococcus, Streptococcal toxic shock syndrome, Disseminated intravascular coagulation, Protein C deficiency

## Abstract

**Background:**

*Streptococcus pyogenes* is an uncommon pathogen of purpura fulminans, and the pathogenesis of *S. pyogenes*-purpura fulminans remains unclear because of paucity of cases. We reported a pediatric case of *S. pyogenes*-purpura fulminans with literature review of the disease.

**Case presentation:**

A 3-year-old boy showed limping, lethargy and acral gangrene within 24 h. A diagnosis of *S. pyogenes*-purpura fulminans was made for bacterial isolation from throat and peripheral blood. Intensive therapy led to a survival with amputation of the left distal metatarsal bone, and normal development. The isolated M12 carried no mutation of *csrS/R* or *rgg*. Thrombophilia or immunodeficiency was excluded.

**Discussion:**

Twelve-reported cases (9 pediatric and 3 elderly) of *S. pyogenes*-purpura fulminans started with shock and coagulopathy. Five patients age < 8 years had no underlying disease and survived. One youngest and two immunocompromised patients died.

**Conclusion:**

*Streptococcus pyogenes*-acute infectious purpura fulminans is a distinctive rare form of aggressive GAS infections.

## Background

*Streptococcus pyogenes* (*S. pyogenes*) is the causative bacteria of scarlet fever, pharyngitis, impetigo, and cellulitis, as well as rheumatic fever and acute poststreptococcal glomerulonephritis [1]. The group A β-hemolytic Streptococcus (GAS) is associated with Sydenham’s chorea and pediatric acute-onset neuropsychiatric syndrome [2]. *S. pyogenes* is isolated from the throat of 10–30% children with acute pharyngitis, and healthy carriers are the source of outbreak. On the other hand, the most aggressive GAS infections comprise streptococcal toxic shock syndrome (TSS) and necrotizing fasciitis (NF) [3]. The invasive GAS infection occurs with high mortality and morbidity rates in both immunocompetent and immunocompromised adults irrespective of age, sex, and ethnicity. The ominous disease may thus account for the virulence of isolated strains. In particular, serotype M3 GAS isolates are known to be associated with severe invasive infection [4].

Purpura fulminans (PF) is a life-threatening condition characterized by sudden-onset progressive cutaneous hemorrhage and acral necrosis [5]. It is classified into neonatal, idiopathic, and acute infectious PFs. Neonatal PF develops within 72 h after birth, presenting with cerebral hemorrhage and/or thrombosis, purpuric skin lesions and symmetrical acral gangrene. It is caused by heritable protein C deficiency [6]. Idiopathic PF occurs 7–10 days after the onset of varicella or streptococcal infection, in the presence of transient autoantibodies directed against protein C and protein S [7]. The most common form, acute infectious PF, occurs superimposed on sepsis due to *Neisseria meningitidis*, *Streptococcus pneumoniae*, *Haemophilus influenzae*, *Staphylococcus aureus*, and rickettsiae [8]. *S. pyogenes* is an uncommon pathogen of PF in adults, and the pathogenesis of severe streptococcal disease remains unclear.

We herein report a rare case of acute infectious PF caused by *S. pyogenes* infection. The isolated strain of GAS from the previously healthy boy was M12 without high-virulent mutations of *csrS/R* or *rgg* [9, 10]. Twelve reviewed cases indicated that *S. pyogenes* is a weighty organism of acute infectious PF, occurring as a distinct type invasive GAS infection affecting children and elderly.

## Materials and methods

### Genetic study for the bacteria

Total DNA was extracted from bacterial cells and the nucleotide sequences of the *csrS*, *csrR*, and *rgg* genes were determined by automated sequencers, such as an Applied Biosystems 3130xl Genetic Analyzer (Applied Biosystems, Tokyo, Japan), as described previously [10]. The molecular characterization of the isolated strain was completed in the National Institute of Infectious Diseases (Tokyo, Japan).

### Genetic and/or functional screening tests for the host

Genomic DNA was extracted from peripheral blood after obtaining informed consent from the patients. For screening of thrombophilia, after coagulation study, the direct sequencing of polymerase chain reaction (PCR) products was completed for the coding regions of protein C gene (*PROC* exons 1–9) and as described previously [11]. For screening of primary immunodeficiency diseases, flow-cytometric analyses were performed to diagnose phagocyte disorders and IRAK4/MyD88 deficiency [12]. Target-sequencing was then performed for the genes including *STAT3*, *PIK3D*, and *PIK3R* by the established method [13].

## Case presentation

A previously healthy 3-year-old boy showed limping with the left leg pain, and then fever 6 h later. Next morning, he was transferred to an emergency hospital because of loss of consciousness and purpuric legs. Within a couple of hours, shock vital signs emerged and ecchymoses extended over the lower extremities. The patient was admitted to a pediatric intensive care unit on cardiopulmonary assist and catecholamine support. He had atopic dermatitis and one history of pneumonia in infancy. The growth and development were normal. There was neither consanguinity nor informative family history.

**Fig. 1:**
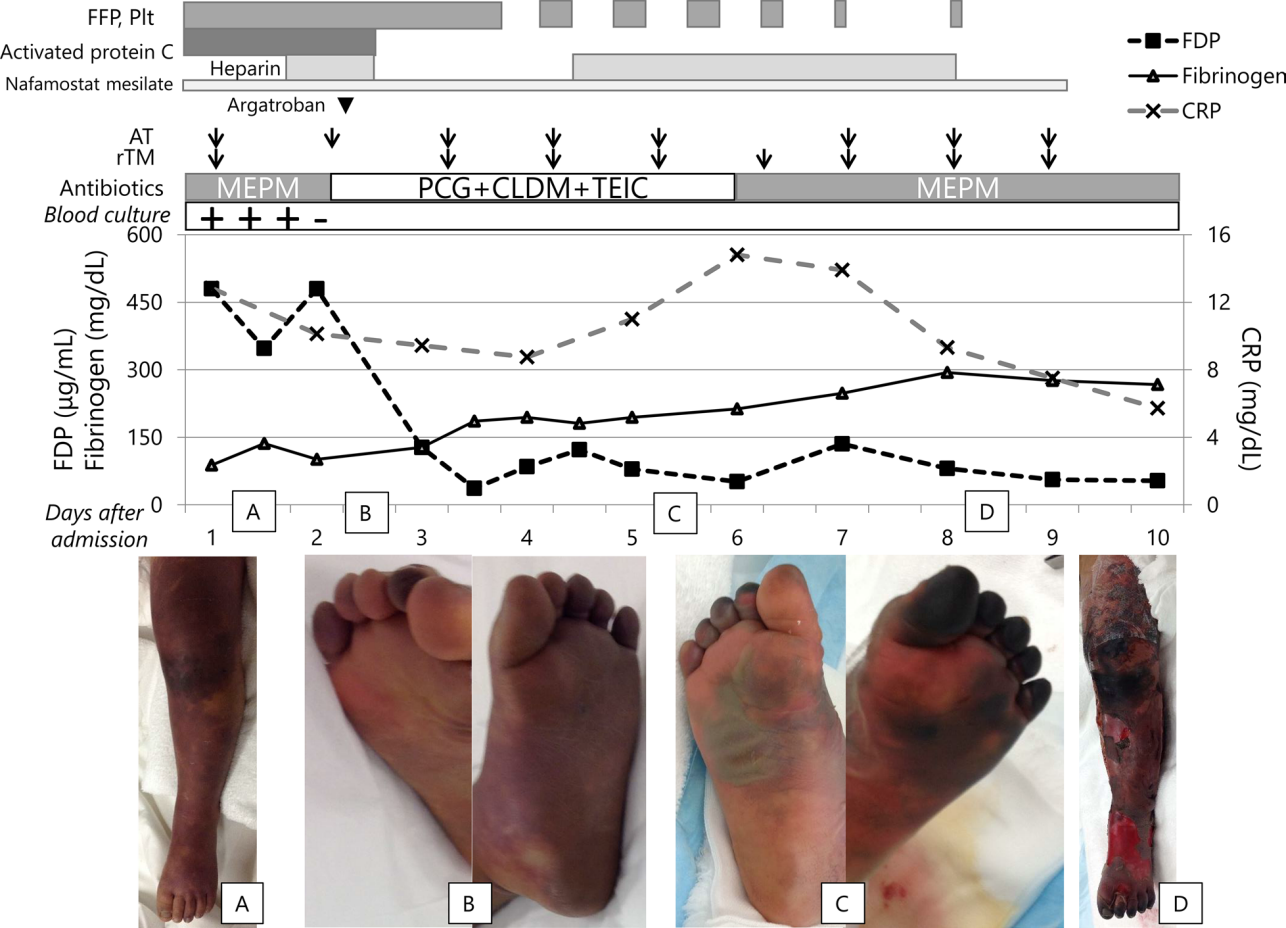
Treatment course of the pediatric patient with *Streptococcus pyogenes*-sepsis purpura fulminans. *FFP* fresh frozen plasma, *Plt* platelet, *FDP* fibrinogen and fibrin degradation products, *CRP* C-reactive protein, *AT* antithrombin, *rTM* recombinant thrombomodulin, *MEPM* meropenem, *PCG* penicillin G, *CLDM* clindamycin, *TEIC* teicoplanin

On admission, the comatose patient showed 180/min of tachycardia and unmeasurable blood pressure on the assist ventilation. Light reflex was prompt. The body temperature was 40.1 °C. Capillary refilling time was prolonged over 2 s, while cardiopulmonary sounds were normal. There was no hepatosplenomegaly or lymphadenopathy. Purpura and ecchymoses expanded to the both legs with necrotic toes (Fig. 1). Petechiae spread over the face, body and upper extremities. Complete blood counts showed a leukocyte count of 0.329 × 10^9^/L with 80% neutrophils, 17% lymphocytes, 3% monocytes, a hemoglobin concentration of 11.0 g/dL, and a platelet count of 3.8 × 10^9^/L. Schizocytosis and hemoglobinuria indicated hemolysis. Serum biochemistries revealed increased levels of blood urea nitrogen (24 mg/dL, reference range [rr]: 8–20), creatinine (0.5 mg/dL, rr: 0.2–0.45), total bilirubin (1.8 mg/dL, rr: 0.3–0.9), aspartate aminotransferase (381 U/L, rr: 24–43), alanine aminotransferase (99 U/L, rr: 9–30), lactate dehydrogenase (1203 U/L, rr: 190–365), creatine kinase (731 U/L, rr: 43–270), and C-reactive protein (12.83 mg/dL, rr: < 0.04). Coagulation studies showed hypofibrinogenemia (139 mg/dL, rr: 200–400), prolonged prothrombin time (PT 21.2 s, control 10–13.5 s) and activated partial thromboplastin time (APTT 53.1 s, control 26–41 s), and increased levels of fibrinogen degradation products (FDP 445.0 μg/mL, rr: 1–10) and D-dimer (142.1 μg/mL, rr: 0.15–1). Thrombin-antithrombin complex (TAT) was > 120.0 ng/mL (rr: < 4), plasmin α2–antiplasmin complex (PIC) was 25.2 μg/mL (rr: < 0.8) and thrombomodulin 37.7 FU/mL (rr: < 4.5). Plasma activities of protein C (13%, rr: 64–131) and protein S (52%, rr: 62–121) were respectively low, and those of antithrombin were normal (85%, rr: 68–130). The rapid antigen tests were positive for *S. pyogenes* and negative for *Pneumococcus*. *S. pyogenes*, determined later as the serotype M12, was isolated from throat and peripheral blood. Sterile cerebrospinal fluid showed no pleocytosis. These determined the diagnosis of acute infectious PF with *S. pyogenes*-septic shock. Antibiotic and anticoagulant therapy was started on the intensive care. Next day, leg purpura and acral gangrene extended. Anticoagulation with plasma-derived activated protein C (Anact C^®^), gabexate mesilate, fresh frozen plasma replacement, and platelet infusion, was intensified by the addition of a direct thrombin inhibitor (argatroban), antithrombin, recombinant thrombomodulin, and low-molecular heparin. The relaxing incisions were then conducted for the compartment syndrome of both legs on the 3rd day of illness. *S. pyogenes* was also isolated from the debridement tissues, and the histopathology indicated absent necrosis of the fascia and necrotic alteration of the muscle without specific inflammation. The continuous infusion of penicillin G led to the negative blood culture 24 h after the start of antibiotic therapy. Under controlled sepsis and coagulopathy, continuous irrigation and debridement were repeated. On the 13th day, debridement tissues showed necrotic muscle and fascia with chronic inflammatory changes. The histological changes indicated that the necrotic muscle tissues originated from ischemia rather than spreading infection of the fascia. The distal portions of the left metatarsal bone were finally amputated. Repeated skin-autografts resulted in the cosmetic and functional improvement of the extremities. There was no evidence of primary immunodeficiency diseases including asplenia, phagocyte disorders, hypogammaglobulinemia, immunoglobulin G subclass deficiency, complement deficiency or IRAK4/MyD88 deficiency, and absent mutation of *STAT3*, *PI3KD*, and *PIK3RI* genes. The genetic study of protein C or Factor V Leiden excluded heritable thrombophilia. He lives an active life for walking rehabilitation with no delayed development at 6 years of age. The isolated strain possessed T12 and M12 antigens, and *emm12.0* gene, but no mutation of *csrS/R* or *rgg*.

Twelve reported patients with acute infectious PF with *S. pyogenes* infection including the present case are shown in Table 1. Nine patients developed at age ≤ 10 years, and the remaining three were ≥ 50 years of age. All but one cases presented with septic shock and DIC, but not necrotizing fasciitis. Four immunocompromised patients were over 6 years. Five children age < 8 years had no underlying disease, and survived. The youngest one and two immunocompromised patients died.

**Table 1:** Reported cases of acute infectious purpura fulminans with *Streptococcus pyogenes* infection

Patient no	Sex	Age at onset	Underlying disease	Plasma activity of protein C (%)	Complications	Outcomes			Reports
							Extremities	Neurological	
1	M	2 months	No	NR	Shock, DIC	Death	NR	Death	Lloyd and Bolte [14]
2	M	4 months	No	6	Shock, DIC	Alive	All toes and 7 digits lost	No deficits	Dhodapkar et al. [15]
3	F	2.5 years	No	NR	Shock, DIC, ARDS, MOF	Alive	Preserved	Brain death	Daskalaki et al. [16]
4	M	3 years	No	13	Shock, DIC	Alive	Amputation	No deficits	Our case
5	M	5 years	No	60	Shock, DIC	Alive	Preserved	No deficits	Dhodapkar et al. [15]
6	M	7 years	No	NR	Shock, DIC	Alive	Amputation	NR	Davis et al. [17]
7	F	7 years	Chylothorax	NR	Shock, DIC, MOF	Alive	Amputation	NR	Cruz et al. [18]
8	F	8 years	JIA, TNFα-blocker	NR	Shock, DIC, Renal failure	Death	Amputation	Death	Renaud et al. [19]
9	F	10 years	JIA, TNFα-blocker	NR	Shock, DIC, ARDS	Alive	Amputation	NR	Lovell et al. [20]
10	M	50 years	No	Normal	Shock, DIC, Renal failure	Alive	Skin grafting	NR	Gupta [21]
11	F	62 years	Asplenia, NHL	NR	DIC	Death	NR	Death	Ward et al. [22]
12	F	72 years	No	NR	Shock, DIC	Alive	NR	NR	Ashokkumar et al. [23]

*DIC* disseminated intravascular coagulation, *ARDS* acute respiratory distress syndrome, *MOF* multiple organ failure, *JIA* juvenile idiopathic arthritis, *TNF* tumor necrosis factor, *NHL* non-Hodgkin’s lymphoma, *NR* not recorded

## Discussion and conclusions

*Streptococcus pyogenes* is involved in both “acute infectious (sepsis-associated) PF” and “idiopathic (antibody-mediated) PF”. Neither of TSS or NF typified the present case. M types 1, 3, 12, and 28 are the common isolates associated with shock and multiorgan failure. However, the isolated strain in this case had no high-virulent mutation. The literature review first revealed no patients age 10–50 years, in contrast to the young adult patients with TTS and NF. No previous reports verified the bacteriological property or host predisposition [21]. Shock and coagulopathy was the hallmark of the disease onset, and half of them were previously healthy children. *S. pyogenes*-acute infectious PF may exemplify a distinct form of invasive GAS infection heralded by septic shock.

**Fig. 2:**
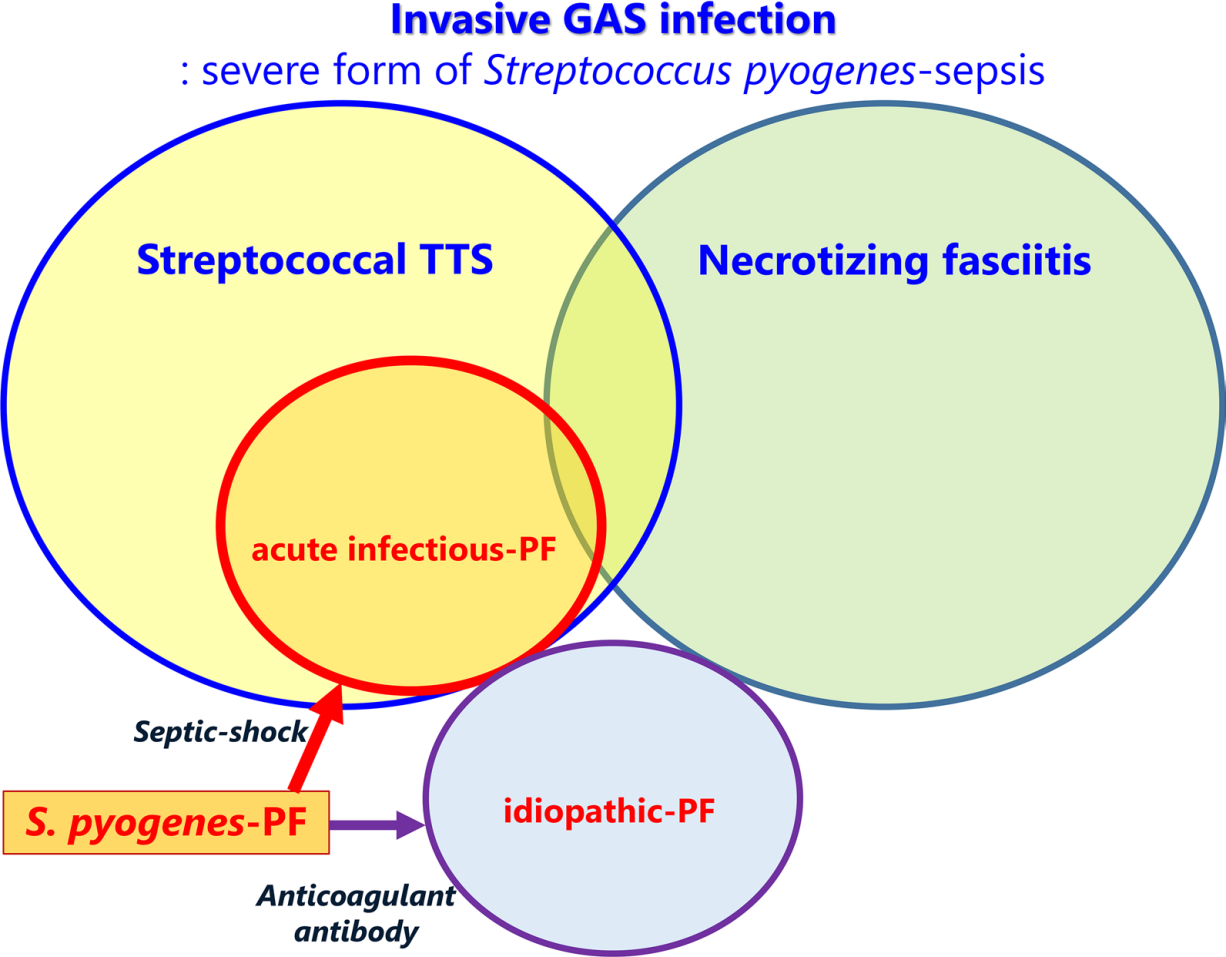
A conceptual diagram of invasive group A Streptococcus infection and *Streptococcus pyogenes*-associated purpura fulminans. GAS, group A Streptococcus; TSS, toxic shock syndrome; PF, purpura fulminans; *S. pyogenes*, *Streptococcus pyogenes*

The pathogenesis of *S. pyogenes* -acute infectious PF remains unclear, because of the paucity of cases. Several toxins released from meningococci or pneumococci trigger the thrombotic action and subsequent hypercoagulability [24]. The streptococcal pyrogenic exotoxins (speA, speB and speC) induce cytotoxicity and pyrogenicity, and also enhance the lethal effects of endotoxins on the host [25]. *S. pyogenes*-derived superantigens cause TSS in healthy adults, but no thrombogenic toxins of GAS have been identified. *S. pyogenes* are considered to evolve the aggressive form via the horizontal crosstalk among the bacteria, although the precise mechanism has not been clarified [26]. The invasive GAS infection of TSS or NF makes a distinction in the clinical expression of patients. NF is an infection of the fascia and multiple layers of soft tissue from epidermis to muscle that characterized by extensive and rapidly spreading necrosis. Molecular studies [27] suggested that the injured muscle cells promote the adhesion of *S. pyogenes* that deteriorate local tissue infection. Streptococcal TSS is defined as any streptococcal infection associated with the sudden onset of shock and organ failure. Cytokine storm is crucial for the development of circulatory failure in bloodstream or deep tissue infection. In addition to the excessive T cell activation via superantigens, *S. pyogenes* exotoxins may induce the production of vasodilator or cardiac depressant leading to shock [28]. We demonstrated the skewed age distribution and the exclusive presentation of shock and coagulopathy at the onset of *S. pyogenes*-PF. A higher frequency of TSS has been noticed in *S. pyogenes*-PF than seen in *S. pyogenes*-NF [29]. The physiological low activity levels of protein C and protein S in infants raise the risk of thrombogenicity in severe infection [30]. It is not to say exaggerated that the rapid aggression of *S. pyogenes*-PF arises from the vulnerability to impaired circulation and hypercoagulability on toxic or, if any, septic shock (Fig. 2). The GAS virulence in *S. pyogenes*-PF may involve the ability to produce circulatory collapse-inducing factors rather than the tissue invasiveness of strain.

Antibiotic and anticoagulant therapy is the mainstay of acute infectious PF. The treatment goal is the survival free from amputation and neurological deficit. Our patient required repeated debridement of the legs to treat compartment syndrome but not for NF. The histopathologic study showed ischemic necrosis in the subcutaneous tissues and muscles. It may corroborate a pivotal role of the circulatory failure in the pathophysiology of *S. pyogenes*-PF. The pathogen was repeatedly isolated from the debridement tissues until the start of continuous PCG infusion with CLDM, that is usually used for the treatment of invasive GAS infection [31]. It accounted for the insufficient drug delivery in the ischemic tissues rather than the strain resistance. Our observations recommend the intensive circulatory support on invasive GAS-directed chemotherapy to minimize the chance of amputation in patients with *S. pyogenes*-PF.

In conclusion, *S. pyogenes*-acute infectious PF rarely occurred as the most severe form of *S. pyogenes*-TSS in healthy young children and the elderly. The first treatment of circulatory failure may be crucial for the outcomes of *S. pyogenes*-PF.

## Abbreviations

PF: purpura fulminans
GAS: group A Streptococcus
TSS: toxic shock syndrome
DIC: disseminated intravascular coagulation
ARDS: acute respiratory distress syndrome
MOF: multiple organ failure
JIA: juvenile idiopathic arthritis
TNF: tumor necrosis factor
NHL: non-Hodgkin’s lymphoma
TSS: toxic shock syndrome
NF: necrotizing fasciitis

## References

[1] Hamada S (2015). Molecular and genomic characterization of pathogenic traits of group A *Streptococcus pyogenes*. Proc Jpn Acad Ser B Phys Biol Sci.

[2] Thienemann M (2017). Clinical management of pediatric acute-onset neuropsychiatric syndrome: part I-psychiatric and behavioral interventions. J Child Adolesc Psychopharmacol.

[3] Rudolph K (2016). Epidemiology of invasive group A streptococcal disease in Alaska, 2001 to 2013. J Clin Microbiol.

[4] Sarkar P (2017). Regulatory gene mutation: a driving force behind group A Streptococcus strain- and serotype-specific variation. Mol Microbiol.

[5] Chalmers E (2011). Purpura fulminans: recognition, diagnosis and management. Arch Dis Child.

[6] Price VE (2011). Diagnosis and management of neonatal purpura fulminans. Semin Fetal Neonatal Med.

[7] Michael L (1995). Postinfectious purpura fulminans caused by an autoantibody directed against protein S. J Pediatr.

[8] Luke N (2017). Widespread subcutaneous necrosis in spotted fever group rickettsioses from the coastal belt of Sri Lanka—a case report. BMC Infect Dis.

[9] Ikebe T (2015). Increased prevalence of group A Streptococcus isolates in streptococcal toxic shock syndrome cases in Japan from 2010 to 2012. Epidemiol Infect.

[10] Ikebe T (2010). Highly frequent mutations in negative regulators of multiple virulence genes in group A streptococcal toxic shock syndrome isolates. PLoS Pathog.

[11] Ichiyama M (2016). Age-specific onset and distribution of the natural anticoagulant deficiency in pediatric thromboembolism. Pediatr Res.

[12] Takada H (2006). Delayed separation of the umbilical cord in two siblings with Interleukin-1 receptor-associated kinase 4 deficiency: rapid screening by flow cytometer. J Pediatr.

[13] Minegishi Y (2007). Dominant-negative mutations in the DNA-binding domain of STAT3 cause hyper-IgE syndrome. Nature.

[14] Lloyd TR, Bolte RG (1986). Rhinocerebral mucormycosis in an infant with streptococcal sepsis and purpura fulminans. Pediatr Infect Dis..

[15] Dhodapkar K, Corbacioglu S, Chang MW (2000). Purpura fulminans caused by group A β-hemolytic Streptococcus sepsis. J Pediatr..

[16] Daskalaki MA, Boeckx WD, DeMey A (2013). Toxic shock syndrome due to group A beta-hemolytic streptococcus presenting with purpura fulminans and limb ischemia in a pediatric patient treated with early microsurgical arteriolysis. J Pediatr Surg..

[17] Davis MD, Dy KM, Nelson S (2007). Presentation and outcome of purpura fulminans associated with peripheral gangrene in 12 patients at Mayo Clinic. J Am Acad Dermatol..

[18] Cruz AT, Creech CB, Smith KP (2005). Streptococcus pyogenes purpura fulminans in a child with congenital chylothorax. Infect Dis Clin Pract..

[19] Renaud C, Ovetchkine P, Bortolozzi P (2011). Fatal group A Streptococcus purpura fulminans in a child receiving TNF-α blocker. Eur J Pediatr..

[20] Lovell DJ, Giannini EH, Reiff A (2003). Long-term efficacy and safety of etanercept in children with polyarticular-course juvenile rheumatoid arthritis: interim results from an ongoing multicenter, open-label, extended-treatment trial. Arthritis Rheum..

[21] Gupta D (2016). Acute infectious purpura fulminans caused by group A β-hemolytic Streptococcus: an uncommon organism. Indian Dermatol Online J.

[22] Ward KM, Celebi JT, Gmyrek R (2002). Acute infectious purpura fulminans associated with asplenism or hyposplenism. J Am Acad Dermatol..

[23] Ashokkumar GK, Madhu R, Shaji M (2015). Bilateral symmetrical digital gangrene of upper and lower limbs due to purpura fulminans caused by Streptococcus pyogenes: A rare entity. Indian J Crit Care Med.

[24] Tunjungputri RN (2017). Phage-derived protein induces increased platelet activation and is associated with mortality in patients with invasive pneumococcal disease. MBio.

[25] Reglinski M (2014). The contribution of group A streptococcal virulence determinants to the pathogenesis of sepsis. Virulence.

[26] Al-Shahib A (2016). Emergence of a novel lineage containing a prophage in emm/M3 group A Streptococcus associated with upsurge in invasive disease in the UK. Microb Genomics.

[27] Bryant AE, Bayer CR, Huntington JD (2006). Group A streptococcal myonecrosis: increased vimentin expression after skeletal-muscle injury mediates the binding of *Streptococcus pyogenes*. J Infect Dis.

[28] Bolz DD, Li Z, McIndoo ER (2015). Cardiac myocyte dysfunction induced by streptolysin O is membrane pore and calcium dependent. Shock.

[29] Sellers BJ, Woods ML, Morris SE (1996). Necrotizing group A streptococcal infections associated with streptococcal toxic shock syndrome. Am J Surg.

[30] Aytemiz G, Deniz A (2001). Outcome of noncatheter-related thrombosis in children: influence of underlying or coexisting factors. J Pediatr Hematol Oncol.

[31] Stevens DL, Bisno AL, Chambers HF (2014). Practice guidelines for the diagnosis and management of skin and soft tissue infections: 2014 update by the infectious diseases society of America. Clin Infect Dis.

